# Elevated body fat percentage is linked to increased risk of diabetes: a longitudinal retrospective cohort study based on Chinese adults

**DOI:** 10.3389/fnut.2025.1510210

**Published:** 2025-06-19

**Authors:** Weicong Pan, Shichun Cai, Zhenhua Huang, Ke Yu

**Affiliations:** ^1^Department of Emergency Medicine, The First Affiliated Hospital of Shenzhen University, Shenzhen Second People’s Hospital, Shenzhen, China; ^2^Department of Quality Control, Pengpai Memorial Hospital, Shanwei, China; ^3^Department of Preventive HealthPengpai Memorial Hospital, Shanwei, China; ^4^The First Affiliated Hospital of Jinan University, Guangzhou, China; ^5^Department of Pulmonary and Critical Care Medicine, The First Affiliated Hospital of Shenzhen University, Shenzhen Second People’s Hospital, Shenzhen, China

**Keywords:** body fat percentage, diabetes risk, nonlinear relationship, retrospective cohort study, Chinese adults

## Abstract

**Objective:**

Previous studies have extensively explored the association between body mass index (BMI) and the risk of diabetes. However, evidence regarding the relationship between body fat percentage (BF%) and diabetes risk remained limited. This study aimed to investigate the association between BF% and the risk of diabetes among Chinese adults.

**Methods:**

We conducted a retrospective cohort study involving 211,833 Chinese adults who underwent health evaluations from 2010 to 2016. The relationship between baseline BF% and diabetes risk was analyzed using Cox proportional hazards regression models. Additionally, cubic spline functions and smooth curve fitting were used to examine the nonlinear relationship between BF% and diabetes onset. Sensitivity and subgroup analyses were performed to validate the robustness of our findings.

**Results:**

After adjusting for the variables, our analysis demonstrated that a 1% increase in BF% is associated with a 1.04-fold higher risk of diabetes (HR: 1.04, 95% CI: 1.04–1.05, *p* < 0.0001). Diabetes risk progressively increased across BF% quartiles (Q1 to Q4), with Q4 showing a significantly higher risk than Q1 (adjusted HR: 2.72, 95% CI: 2.19–3.37). Furthermore, a nonlinear association between BF% and diabetes risk was identified, with a critical inflection point at 25.09%. Below this threshold, the HR was 1.17 (95% CI: 1.13–1.21), while above it, the HR was 1.02 (95% CI: 1.02–1.03). The subgroup analysis and sensitivity analysis demonstrated the robustness of these results.

**Conclusion:**

This study indicates a positive, nonlinear relationship between BF% and diabetes risk in Chinese adults. Reducing BF% below the identified threshold could significantly lower the risk of developing diabetes.

## Introduction

Diabetes is a chronic metabolic disorder affecting multiple organ systems and leading to significant health complications (1). In 2021, the International Diabetes Federation (IDF) estimated that approximately 536.6 million adults worldwide had diabetes, with this number expected to rise to 783.2 million by 2045 (2). Diabetes has become a major global health crisis, contributing to widespread morbidity, mortality, and a substantial economic burden (3). In 2017, diabetes caused approximately 5 million deaths worldwide, and global healthcare expenditure on diabetes was estimated at USD 850 billion. It is estimated that nearly half (49.7%) of all people with diabetes are undiagnosed (4). Therefore, preventing diabetes through early diagnosis and treatment, along with exploring factors influencing prognosis, has become a critical research focus in recent years.

Type 2 diabetes mellitus (T2DM) risk factors include overweight or obesity, physical inactivity, family history of diabetes, and unhealthy dietary habits (5, 6). Traditional measures like body mass index (BMI) often fall short in accurately assessing body fat and its distribution, particularly in individuals with normal weight but high body fat (7). Body fat percentage (BF%) is calculated based on weight, age, and gender. It represents the proportion of fat relative to total body weight and provides a more precise evaluation of metabolism and inflammation (8). Unlike BMI, BF% directly measures body fat, offering deeper insights into body composition (9). Its safety, simplicity, and affordability have made BF% increasingly popular in both home settings and medical check-ups. Emerging evidence suggests that compared BMI, BF% is more strongly associated with cardiovascular disease risk factors, including diabetes mellitus, hypertension, and dyslipidemia (10, 11). Recent studies have shown a positive correlation between BF% and diabetes (12, 13). A survey-based study in China revealed that exceeding specific thresholds of BF% and trunk fat percentage significantly increases the risk of developing T2DM (14). Additionally, research conducted in Mexican populations indicates that BF% may be a better predictor of T2DM than BMI (12).

However, current research is primarily limited to cross-sectional studies with relatively small sample sizes and may not account for ethnic differences. Moreover, there is a lack of large-scale, long-term cohort studies in Chinese populations. Therefore, this study aims to investigate whether BF% is independently related to the risk of incident diabetes in a large cohort population across 32 sites and 11 cities in China. In this study, we conducted a secondary data analysis using previously published data to explore the correlation between BF% and the risk of incident diabetes.

## Methods

### Study design

This study adopted a retrospective cohort design to analyze the data. The data originated from a prior cohort study by Chinese scientists, documented by Chen et al. (15). Baseline BF% served as the primary independent variable. The outcome variable, defined as the presence of diabetes mellitus (DM), was dichotomous: 0 for non-diabetic and 1 for diabetic individuals.

### Data source

Data were obtained from the DATADRYAD repository,^1^ provided by Chen, Ying et al. The data is freely accessible under the Creative Commons Attribution-Non-Commercial (CC BY-NC 4.0) license, which allows for non-commercial sharing, modification, remixing, and creation of derivative works, provided that appropriate credit is given to the original authors and source.

According to Dryad’s guidelines, researchers may use this data for secondary analyses without infringing on the rights of the original authors. Therefore, ethical approval was not required for this secondary analysis. The original study adhered to the Declaration of Helsinki, ensuring all procedures complied with its protocols and rules. These same standards were upheld during the secondary analysis.

### Study population

**Figure 1:**
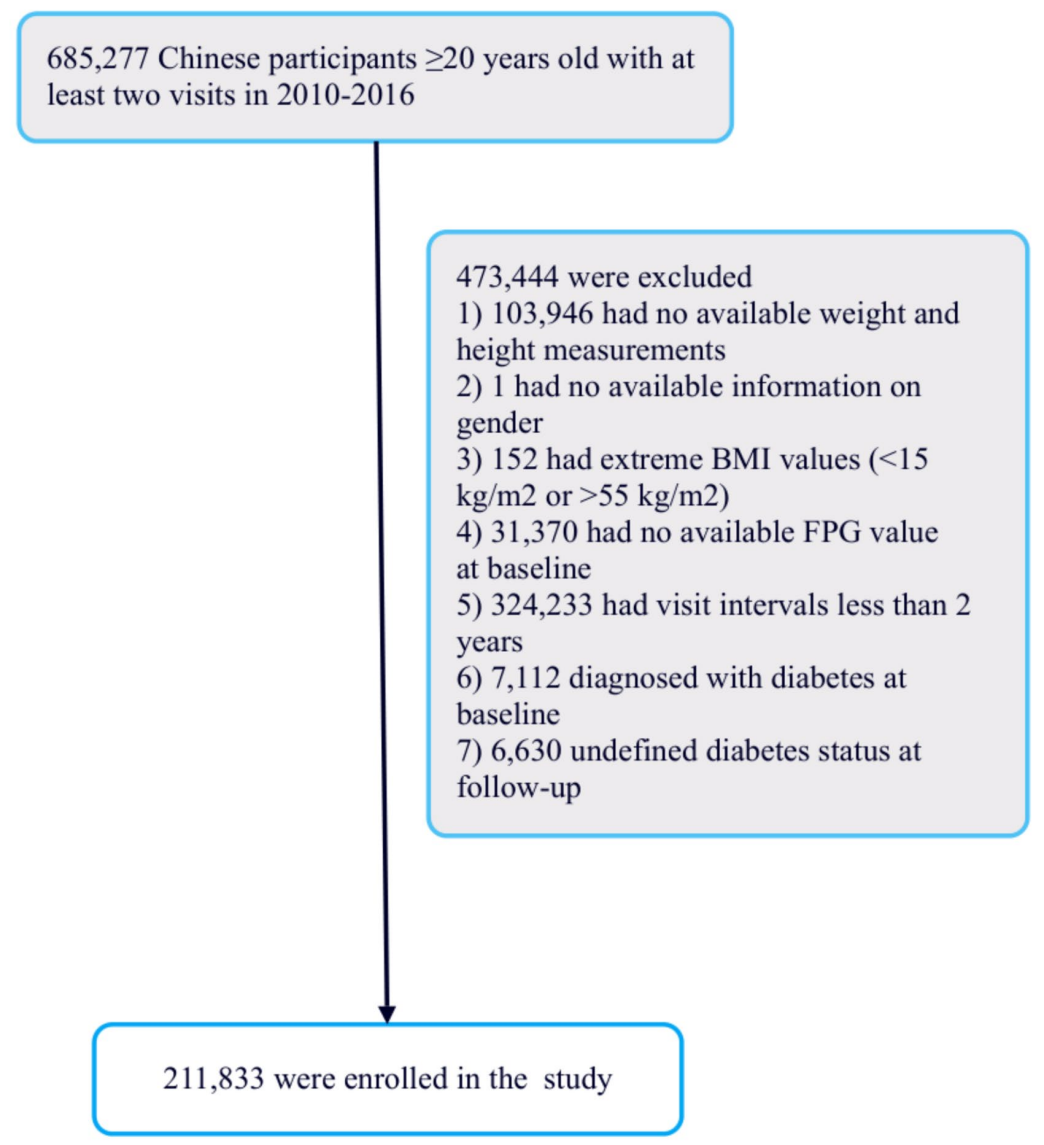
Flowchart of study participants.

The scientists initially utilized a digital database created by the Rich Healthcare Group in China, containing medical records from health examinations conducted between 2010 and 2016, across 32 regions and 11 cities nationwide (15). The study initially included 685,277 participants, all aged 20 or older, who had undergone at least two health examinations. After excluding 473,744 individuals, 211,833 participants remained for analysis (Figure 1).

### Data collection

In the initial study, researchers used standardized questionnaires to collect baseline data, which included demographic information such as age and gender, lifestyle habits like smoking and alcohol consumption, and family history of diabetes. BMI was calculated by dividing weight (in kilograms) by height (in meters) squared. Blood pressure was measured with standard mercury sphygmomanometers. Fasting venous blood samples were drawn after a minimum of 10 h of fasting at each visit. Plasma glucose, triglycerides (TG), total cholesterol (TC), high-density lipoprotein cholesterol (HDL-c), blood urea nitrogen (BUN), serum creatinine (Scr), and low-density lipoprotein cholesterol (LDL-c) were analyzed using an autoanalyzer (Beckman 5,800).

### Variables and outcome measures

BF% was recorded as a continuous variable. It was calculated using the formula: BF% = (1.20 × BMI) + (0.23 × Age) − (10.8 × Gender) − 5.4, where Gender is coded as 1 for males and 0 for females (16, 17). Diabetes was diagnosed based on fasting plasma glucose levels of ≥7.00 mmol/L (18) or self-reported diabetes during follow-up. Follow-up data were censored at the earlier of the two dates: diabetes diagnosis or last visit (15). The outcome variable, incident diabetes, was defined as a binary variable (0 = non-MD, 1 = MD).

### Covariates

The selection of covariates was guided by our clinical experience and relevant findings from existing literature. We included several variables in our analysis as covariates. The first category comprises continuous variables, including age, height, weight, BMI, ALT, SBP, DBP, BUN, Scr, HDL-c, LDL-c, TG, and TC. The second category includes categorical variables such as gender, family history of diabetes, alcohol consumption, and smoking habits.

### Statistical analysis

Participants were divided into four groups based on BF% quartiles. Continuous variables were presented as mean ± standard deviation for normally distributed data, and as median with interquartile range for non-normally distributed data. Categorical variables were expressed as frequencies (percentages). For group comparisons, the chi-square test was used to analyze differences in categorical variables. One-way ANOVA was applied to continuous variables with normal distribution, while the Kruskal-Wallis H test was used for non-normally distributed continuous variables. Missing data is inevitable in observational studies. We have defined the missing data for smoking and alcohol as unknown. For other missing data, we have performed multiple imputations.

Univariate and multivariate Cox proportional hazards models were employed to analyze the association between BF% and diabetes risk. After assessing multicollinearity, three models were considered: an unadjusted basic model, a partially adjusted model (Model I, adjusting for BMI), and a fully adjusted model (Model II, adjusting for BMI, SBP, DBP, ALT, TC, TG, HDL-c, LDL-c, BUN, Cr, smoking status, alcohol consumption, family history of diabetes, and baseline FPG). Hazard ratios (HR) and 95% confidence intervals (CI) were calculated.

Confounding factors were identified based on clinical experience, literature review, and univariate analysis results, along with multicollinearity screening data. To address non-linear relationships, Cox regression models with cubic spline functions and smooth curve fitting were used, followed by a two-piecewise Cox regression model to further clarify the non-linear association between BF% and diabetes risk. The log-likelihood ratio test was employed to identify the model best suited to explain the relationship between BF% and diabetes risk.

To ensure the robustness of the results, stratified Cox regression models were applied for subgroup analyses and sensitivity analyses. The generalized additive model (GAM) was also used to incorporate continuous covariates as curves (19). All results were reported according to the STROBE guidelines (20, 21). Data analysis was performed using R software and Empower Stats, with statistical significance defined as a two-sided *p*-value of less than 0.05.

## Results

### Characteristics of participants

Table 1 displays the demographic and clinical characteristics of the study participants. The mean age of participants was 42.10 ± 12.65 years, and 116,123 participants (54.82%) were male. The mean follow-up period was 3.12 years. BF% ranged from 7.59 to 66.89, with a mean of 26.25 (refer to Supplementary Figure 1). Nevertheless, 4,174 people, accounting for 1.97%, were ultimately diagnosed with diabetes. Diabetes rates increased progressively across BF% quartiles. Table 1 also provides additional details on the initial traits of participants, categorized by BF% quartiles. Increasing BF% from Q1 to Q4 was significantly associated with higher age, BMI, suggesting a correlation between higher body fat and these factors. The proportion of women increased significantly in the higher quartiles, whereas the proportions of smokers and alcohol consumers were greater in the lower quartiles (*p* < 0.001). Biochemical markers, including TC and LDL-c, FBG, exhibited a progressive increase from Q1 to Q4, whereas Scr levels demonstrated a declining trend (*p* < 0.001). An elevated BF% was linked to a greater number of people with a familial history of diabetes (p < 0.001), indicating a potential rise in diabetes risk (see Table 1).

**Table 1:** The baseline characteristics of participants.

BF% (quartile)	Q1 (7.59–21.73)	Q2 (21.73–26.01)	Q3 (26.01–30.39)	Q4 (30.39–66.89)	*P*-value
Participants	52,945	52,919	52,953	53,016	
Age (years)	34.35 ± 7.12	39.38 ± 10.64	42.94 ± 12.14	51.70 ± 12.98	<0.001
BMI (kg/m^2^)	21.70 ± 2.20	22.84 ± 3.38	23.34 ± 3.38	25.06 ± 3.33	<0.001
SBP (mmHg)	118.26 ± 13.25	117.56 ± 15.49	117.12 ± 16.97	123.31 ± 18.57	<0.001
DBP (mmHg)	73.13 ± 9.17	74.22 ± 10.74	73.64 ± 11.57	75.72 ± 11.43	<0.001
FBG (mg/dL)	4.83 ± 0.56	4.88 ± 0.61	4.91 ± 0.62	5.04 ± 0.63	<0.001
TC (mmol/L)	4.48 ± 0.83	4.69 ± 0.88	4.68 ± 0.88	4.97 ± 0.94	<0.001
TG (mmol/L)	1.20 ± 0.77	1.41 ± 1.15	1.32 ± 1.15	1.43 ± 1.00	<0.001
HDL-c (mmol/L)	1.33 ± 0.28	1.36 ± 0.32	1.39 ± 0.32	1.40 ± 0.31	<0.001
LDL-c (mmol/L)	2.64 ± 0.63	2.74 ± 0.67	2.73 ± 0.67	2.93 ± 0.71	<0.001
ALT (U/L)	19.40 (14.20-28.00)	19.70 (13.00-31.20)	16.10 (11.60-26.20)	17.00 (13.00-24.70)	<0.001
BUN (mmol/L)	4.78 ± 1.12	4.63 ± 1.19	4.54 ± 1.20	4.67 ± 1.22	<0.001
Scr (μmol/L)	78.91 ± 11.43	72.11 ± 15.16	66.87 ± 16.47	62.42 ± 14.69	<0.001
Gender, n (%)					<0.001
Male	51,763 (97.77%)	34,644 (65.47%)	21,736 (41.05%)	7,980 (15.05%)	
Female	1,182 (2.23%)	18,275 (34.53%)	31,217 (58.95%)	45,036 (84.95%)	
Smoking status, n (%)					<0.001
Current smoker	4,174 (7.88%)	4,070 (7.69%)	2,888 (5.45%)	943 (1.78%)	
Ever smoker	1,207 (2.28%)	772 (1.46%)	421 (0.80%)	159 (0.30%)	
Never	13,045 (24.64%)	11,144 (21.06%)	10,576 (19.97%)	10,831 (20.43%)	
Unknown	34,519 (65.20%)	36,933 (69.79%)	39,068 (73.78%)	41,083 (77.49%)	
Drinking status, n (%)					<0.001
Current drinker	319 (0.60%)	468 (0.88%)	405 (0.76%)	159 (0.30%)	
Ever drinker	3,770 (7.12%)	2,831 (5.35%)	1,647 (3.11%)	708 (1.34%)	
Never	14,337 (27.08%)	12,687 (23.97%)	11,833 (22.35%)	11,066 (20.87%)	
Unknown	34,519 (65.20%)	36,933 (69.79%)	39,068 (73.78%)	41,083 (77.49%)	
Family history of diabetes, n (%)			<0.001		<0.001
No	52,280 (98.74%)	51,990 (98.24%)	51,744 (97.72%)	51,475 (97.09%)	
Yes	665 (1.26%)	929 (1.76%)	1,209 (2.28%)	1,541 (2.91%)	
Follow-up (year)	3.11 ± 0.93	3.13 ± 0.94	3.15 ± 0.95	3.11 ± 0.93	<0.001
Incident of DM, n (%)					<0.001
No	52,738 (99.61%)	52,144 (98.54%)	51,673 (97.58%)	51,104 (96.39%)	
Yes	207 (0.39%)	775 (1.46%)	1,280 (2.42%)	1,912 (3.61%)	

Continuous variables were summarized as mean (SD) or medians (quartile interval); categorical variables were displayed as percentage (%). BF%, body fat percentage; BMI, body mass index; SBP, systolic blood pressure; DBP; diastolic blood pressure; TC, total cholesterol; TG triglyceride; HDL-c, high-density lipoprotein cholesterol; ALT, alanine aminotransferase; FBG, fasting plasma glucose; DM, diabetes mellitus. The gray shading is highlights the four groups of participants divided based on body fat percentage (BF%) quartiles. Specifically, Q1 to Q4 represent the first to fourth quartiles.

### The frequency of diabetes among individuals

**Figure 2:**
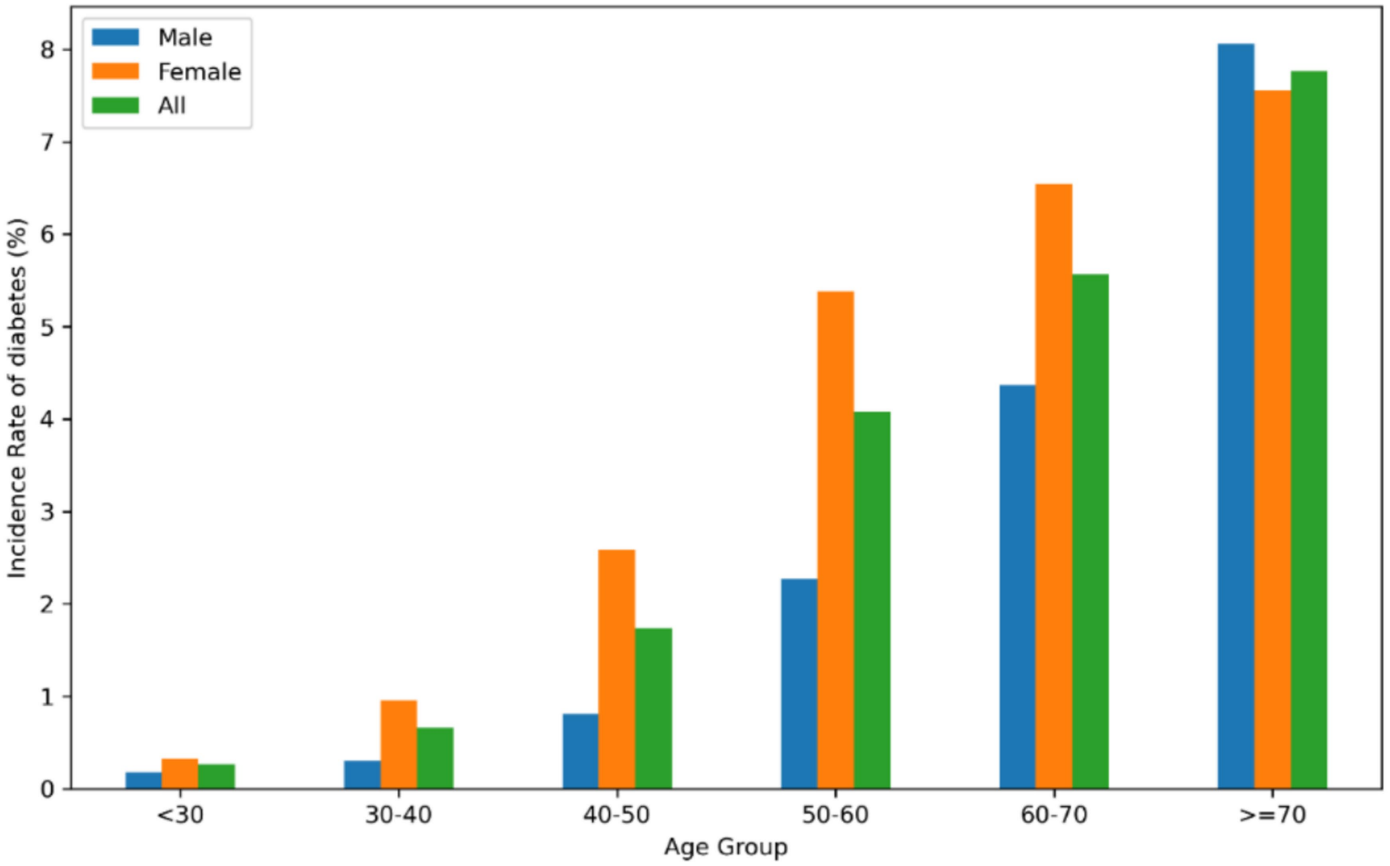
The occurrence of diabetes among Chinese individuals, categorized by age in 10-year intervals. The data indicates that female had a higher rate of developing diabetes compared to man less than 70 years old. The study revealed that as people aged, the rate of diabetes rose in both genders.

According to Table 2, the total cumulative incidence of diabetes was 1.97%. Diabetes incidence rates significantly increase across BF% quartiles, showing a clear upward trend (P for trend < 0.001). Q1 had the smallest incidence rate, recorded at 207 per 10,000 person-years, and a reversal rate of 0.39% (95% CI 0.34–0.44). In contrast, Q2, Q3 and Q4 showed much higher incidence rates, 1.46, 2.42 and 3.61, respectively. The results suggest a significant link between increased BF% and a higher likelihood of developing diabetes, emphasizing the need for effective body fat control to prevent diabetes incidence. In every 10-year age bracket, the occurrence of diabetes rose with advancing age in both males and females. Furthermore, the rate of diabetes development was greater in women compared to men for less than 70 years old (Figure 2). Kaplan–Meier plots illustrate the likelihood of diabetes among different BF% quartiles, highlighting notable variations in progression rates (log-rank test, *p* < 0.001) (see Supplementary Figure 2).

**Table 2:** The Incidence rate of diabetes (% or Per 10,000 person-year).

(BF %)	Participants (n)	Diabetes events (n)	Incidence rate (95% CI) (%)	Per 10,000 person-year
Total	211,833	4,174	1.97 (1.91–2.03)	63.141
Q1	52,945	207	0.39 (0.34–0.44)	12.54
Q2	52,919	775	1.46 (1.36–1.57)	46.645
Q3	52,953	1,280	2.42 (2.29–2.55)	76.825
Q4	53,016	1912	3.61 (3.45–3.77)	116.077
*P* for trend				<0.001

BF%, body fat percentage; CI, confidence interval.

### Examining the elements affecting diabetes risk in individuals using univariate Cox proportional hazards regression analysis

In Table 3, univariate Cox proportional hazards regression identifies key factors influencing diabetes risk. Age is a significant factor, with each additional year increasing the risk by 7% (HR: 1.07, *p* < 0.0001), and males having a 106% higher risk than females (*p* < 0.0001). Metabolic indicators such as BMI, SBP and DBP, and lipid levels (TC and TG) also significantly elevate risk, underscoring their importance in metabolic health. Notably, FBG emerges as the most powerful predictor, with an HR of 10.45, highlighting its critical role in diabetes risk assessment. Lifestyle factors indicate that never smokers have a significantly reduced risk of diabetes (HR: 0.44, *p* < 0.0001), while former drinkers also show a lower risk compared to current drinkers (HR: 0.46, *p* < 0.0001). Additionally, having a family history of diabetes increases the risk by 73% (*p* < 0.0001), highlighting the role of genetic predisposition.

**Table 3:** Univariate Cox proportional hazards regression was used to examine the factors influencing diabetes.

Variable	Characteristics	HR (95% CI) *P*-value
Age (years)	42.10 ± 12.65	1.07 (1.06, 1.07) < 0.0001
Gender, n (%)		
Female	95,710 (45.18%)	1.0
Male	116,123 (54.82%)	2.06 (1.93, 2.20) < 0.0001
BMI (kg/m^2^)	23.24 ± 3.34	1.24 (1.23, 1.25) < 0.0001
SBP (mmHg)	119.06 ± 16.38	1.04 (1.04, 1.04) < 0.0001
DBP (mmHg)	74.18 ± 10.81	1.05 (1.04, 1.05) < 0.0001
TC (mmol/L)	4.71 ± 0.90	1.43 (1.39, 1.47) < 0.0001
TG (mmol/L)	1.34 ± 1.03	1.26 (1.25, 1.28) < 0.0001
HDL-c (mmol/L)	1.37 ± 0.31	0.60 (0.53, 0.68) < 0.0001
LDL-c (mmol/L)	2.77 ± 0.68	1.35 (1.28, 1.42) < 0.0001
ALT (U/L)	23.95 ± 22.13	1.00 (1.00, 1.00) < 0.0001
BUN (mmol/L)	4.66 ± 1.19	1.24 (1.21, 1.26) < 0.0001
Scr (mmol/L)	70.07 ± 15.80	1.01 (1.01, 1.01) < 0.0001
BF (%)	26.24 ± 6.65	1.10 (1.10, 1.11) < 0.0001
FBG (mmol/L)	4.92 ± 0.61	10.45 (10.00, 10.91) < 0.0001
Smoking status, n (%)		
Current smoker	12,075 (5.70%)	1.0
Ever smoker	2,559 (1.21%)	0.79 (0.62, 1.00) 0.0544
Never	45,596 (21.52%)	0.44 (0.39, 0.49) < 0.0001
Unknown	151,603 (71.57%)	0.58 (0.52, 0.64) < 0.0001
Drinking status, n (%)		
Current drinker	1,351 (0.64%)	1.0
Ever drinker	8,956 (4.23%)	0.47 (0.34, 0.65) < 0.0001
Never	49,923 (23.57%)	0.46 (0.35, 0.62) < 0.0001
Unknown	151,603 (71.57%)	0.49 (0.37, 0.65) < 0.0001
Family history of diabetes, n (%)		
No	207,489 (97.95%)	1.0
Yes	4,344 (2.05%)	1.73 (1.48, 2.01) < 0.0001

### The relationship between the BF% and the diabetes was analyzed using a multivariate Cox proportional hazards regression model

Table 4 analyzes the relationship between BF% and diabetes risk using different regression models. After analyzing the imputed data, we found that the results were consistent with those before imputation, which indicates that the conclusions of our study are very robust. Finally, we chose to present the unimputed real data in the manuscript (Supplementary Table S1). In the basic model, without adjusting for covariates, a 1% increase in BF% is associated with a 1.10-fold higher likelihood of diabetes onset (HR: 1.10, 95% CI: 1.10–1.11, *p* < 0.0001). After adjusting for BMI in Model I, the risk remains substantial, with a 1.05-fold increase (HR: 1.05, 95% CI: 1.05–1.06, *p* < 0.0001). Further adjustments in Model II, which account for factors such as liver enzymes, lipid profiles, renal function markers, lifestyle factors, and FPG, show a 1.04-fold higher risk (HR: 1.04, 95% CI: 1.03–1.05, *p* < 0.0001).

**Table 4:** Association between BF% and diabetes risk across various models.

Exposure	Crude model (HR, 95%CI) *P*	Model I (HR, 95%CI) *P*	Model II (HR, 95%CI) *P*	Model III (HR, 95%CI) *P*
BF%	1.10 (1.10, 1.11) < 0.0001	1.05 (1.05, 1.06) < 0.0001	1.04 (1.03, 1.05) < 0.0001	1.05 (1.04, 1.06) < 0.0001
(BF quartiles)				
Q1	Ref	Ref	Ref	Ref
Q2	3.65 (3.13, 4.25) < 0.0001	2.69 (2.31, 3.14) < 0.0001	1.84 (1.48, 2.28) < 0.0001	1.83 (1.47, 2.28) < 0.0001
Q3	5.90 (5.09, 6.83) < 0.0001	3.89 (3.35, 4.52) < 0.0001	2.38 (1.93, 2.93) < 0.0001	2.47 (1.98, 3.07) < 0.0001
Q4	9.34 (8.09, 10.78) < 0.0001	4.51 (3.88, 5.23) < 0.0001	2.72 (2.19, 3.37) < 0.0001	2.97 (2.37, 3.73) < 0.0001
*P* for trend	<0.0001	<0.0001	<0.0001	<0.0001

In the basic model, we did not account for additional variables. Model I: We adjusted BMI. In Model II, we adjusted baseline values for BMI, SBP, DBP, ALT, TC, TG, HDL-c, LDL-c, BUN, Cr, smoking and drinking habits, family history of diabetes, and FPG. In Model III, we adjusted baseline values for BMI (smooth), SBP (smooth), DBP (smooth), ALT (smooth), TC (smooth), TG (smooth), HDL-c (smooth), LDL-c (smooth), BUN (smooth), Cr (smooth), smoking and drinking habits, family history of diabetes, and FPG (smooth). HR stands for Hazard Ratios, CI denotes confidence intervals, and Ref means reference.

Additionally, we applied a Generalized Additive Model (GAM) in Model III, incorporating the continuous variable as a curve. The results remained stable, with an HR of 1.05 (95% CI: 1.04–1.06, *p* < 0.0001), similar to the fully adjusted model. Across BF% quartiles, there is a progressive increase in diabetes risk, with Q4 in Model II showing a 2.97-fold higher risk (HR: 2.97, 95% CI: 2.23–3.73, *p* < 0.0001) compared to Q1. This trend is also observed in Q2 and Q3, with HRs of 1.83 and 2.47, respectively (P for trend all < 0.0001).

### Sensitivity analysis

Table 5 presents the outcomes of sensitivity analyses examining the association between BF% and diabetes risk using three models that each exclude certain participant groups to assess consistency. Initially, BF% was converted from a continuous to a categorical measure based on quartiles and then reintroduced into the regression models.

**Table 5:** Relationship between BF% and risk of diabetes in different sensitivity analyses.

Exposure	Model I (HR, 95%CI) *P*	Model II (HR, 95%CI) *P*	Model III (HR, 95%CI) *P*
BF %	1.05 (1.03, 1.06) < 0.0001	1.04 (1.03, 1.05) < 0.0001	1.05 (1.04, 1.05) < 0.0001
(BF% quartiles)			
Q1	Ref	Ref	Ref
Q2	1.81 (1.43, 2.29) < 0.0001	1.63 (1.30, 2.05) < 0.0001	1.75 (1.39, 2.20) < 0.0001
Q3	1.89 (1.48, 2.41) < 0.0001	2.23 (1.78, 2.80) < 0.0001	2.41 (1.93, 3.01) < 0.0001
Q4	2.28 (1.77, 2.95) < 0.0001	2.49 (1.97, 3.14) < 0.0001	2.79 (2.22, 3.51) < 0.0001
*P* for trend	<0.0001	<0.0001	<0.0001

The model I involved a sensitivity analysis conducted after removing participants with BMI of 25 kg/m^2^ and above (*N* = 60,830). We adjusted BMI, SBP, DBP, ALT, TC, TG, HDL-c, LDL-c, BUN, Cr, smoking habits, alcohol consumption, diabetes family history, and FPG at baseline. Model II involved a sensitivity analysis conducted after removing participants with systolic blood pressure of 140 mmHg or higher (*N* = 21,403). We adjusted BMI, SBP, DBP, ALT, TC, TG, HDL-c, LDL-c, BUN, Cr, smoking habits, alcohol consumption, diabetes family history, and FPG at baseline. Model III involved a sensitivity analysis conducted after removing participants with a diastolic blood pressure of 90 mmHg or higher (*N* = 17,063). We adjusted BMI, SBP, DBP, ALT, TC, TG, HDL-c, LDL-c, BUN, Cr, smoking habits, alcohol consumption, diabetes family history, and FPG at baseline. HR stands for Hazard Ratios, CI denotes confidence intervals, and Ref means reference.

In Model I, which excluded individuals with BMI ≥ 25 kg/m^2^, a 1% increase in BF% was linked to a 5% higher risk of diabetes onset (HR: 1.05, 95% CI: 1.03–1.06, *p* < 0.0001). The analysis of BF% quartiles revealed that participants in the second quartile (Q2) had an 81% higher risk compared to the first quartile (Q1). The risk increased substantially in the third (HR: 1.89, *p* < 0.0001) and fourth quartiles (HR: 2.28, *p* < 0.0001). Model II, which excluded individuals with SBP of 140 mmHg or higher, showed similar results. A 1% rise in BF% was associated with a 4% higher risk of diabetes (HR: 1.04, 95% CI: 1.03–1.05, *p* < 0.0001). In this model, participants in Q4 had a 2.49 times higher risk compared to those in Q1. Model III, which excluded participants with DBP of 90 mmHg or higher, revealed an 5% increased risk per 1% rise in BF% (HR: 1.05, 95% CI: 1.04–1.05, *p* < 0.0001). In this model, Q4 participants had more than twice the risk of diabetes compared to Q1 (HR: 2.79, *p* < 0.0001), with a consistent trend across all quartiles (P for trend < 0.0001).

### Cox regression model utilizing cubic splines to handle non-linear relationships

**Figure 3:**
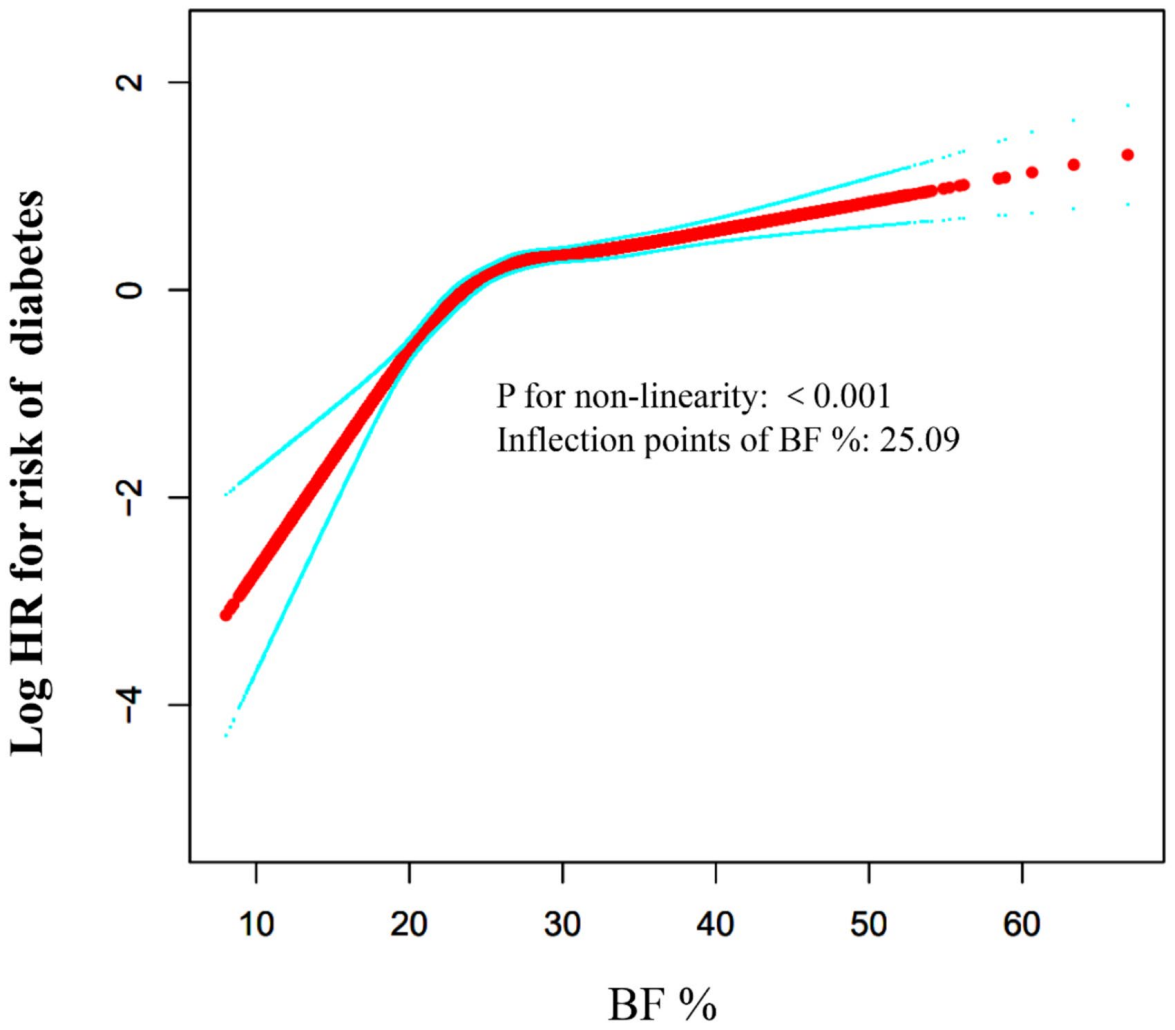
The non-linear relationship between BF % and risk of diabetes. The relationship between BF % and risk of diabetes was non-linear, with the inflection point of BF % being 25.09%.

Using the Cox proportional hazards regression model with cubic spline functions, we observed that the association between BF% and the onset of diabetes was non-linear (Figure 3). Consequently, a segmented Cox proportional hazards regression model was applied to identify two distinct slopes. A conventional binary two-piece Cox proportional hazards regression model was also used for sensitivity analysis, with the optimal model determined via the log-likelihood ratio test.

Table 6 shows the results of the segmented linear regression model, clarifying the association between BF% and diabetes risk, and the threshold point. The analysis found that for every 1% increase in BF%, diabetes risk rises by 4% (HR: 1.04, 95%CI: 1.03–1.05, *p* < 0.0001). Segmented regression analysis identified a BF% inflection point at 25.09%. Below this threshold, even small increases in BF% significantly increase diabetes risk (HR: 1.17, 95%CI: 1.13–1.21, *p* < 0.0001). Above it, the risk remains elevated but grows more slowly (HR: 1.02, 95%CI: 1.02–1.03, *p* < 0.0001). To explore sex - specific differences in the inflection point, stratified analysis found male and female breakpoints at 24.60 and 34.93%, respectively, with similar trends.

**Table 6:** The result of the two-piecewise linear regression model.

Outcome: diabetes	HR, 95%CI *P*-value (all)	HR, 95%CI *P* –value (male)	HR, 95%CI *P* -value (female)
Standard Cox regression	1.04 (1.03, 1.05) < 0.0001	1.15 (1.13, 1.17) < 0.0001	1.21 (1.17, 1.25) < 0.0001
Two-piecewise Cox regression			
Inflection points of BF%	25.09	24.60	34.93
<K	1.17 (1.13, 1.21) < 0.0001	1.32 (1.26, 1.38) < 0.0001	1.30 (1.23, 1.36) < 0.0001
≥K	1.02 (1.02, 1.03) < 0.0001	1.10 (1.08, 1.13) < 0.0001	1.17 (1.13, 1.21) < 0.0001
*P* for log-likelihood ratio test	<0.001	<0.001	0.016

We accounted for baseline measurements of BMI, SBP, DBP, ALT, TC, TG, HDL-c, LDL-c, BUN, Cr, smoking habits, alcohol consumption, diabetes family history, and FPG at baseline. K stands for inflection point. HR stands for hazard ratios, while CI denotes confidence intervals.

### The results of subgroup analyses

Table 7 presents the stratified association between BF% and diabetes risk, considering age, BMI, gender, blood pressure levels, and family history of diabetes. For individuals under 60 years of age, each 1% increase in BF% was associated with a 3% higher diabetes risk (HR: 1.03). In contrast, for those aged 60 and above, the risk increased by 1% (HR: 1.01), indicating that younger individuals are more sensitive to increases in BF%. The hazard ratios were 1.05 for those with a BMI under 25 kg/m^2^ and 1.04 for those with a BMI of 25 kg/m^2^ or higher, indicating that BF% is an independent risk factor regardless of BMI. Women had a higher diabetes risk (HR: 1.19) than men (HR: 1.16), indicating greater susceptibility to the metabolic consequences of increased BF%. The hazard ratios were 1.05 for those with a SBP under 140 mmHg and 1.03 for those with a SBP of 140 mmHg or higher. Similarly, the hazard ratios were 1.05 for those with a DBP under 90 mmHg and 1.01 for those with a DBP of 90 mmHg or higher.

**Table 7:** Stratified associations between BF% and diabetes risk, categorized by age, gender, BMI, SBP, DBP and family diabetes history.

Variable	HR (95% CI)	*P*-value	P for interaction
Age, yeas			<0.0001
<60	1.03 (1.02, 1.05)	<0.0001	
≥60	1.01 (1.00, 1.02)	0.1592	
BMI (kg/m^2^)			0.0613
<25	1.05 (1.04, 1.07)	<0.0001	
≥25	1.04 (1.03, 1.05)	<0.0001	
Gender			<0.0001
Female	1.19 (1.17, 1.21)	<0.0001	
Male	1.16 (1.14, 1.17)	<0.0001	
SBP (mmHg)			0.0229
<140	1.05 (1.04, 1.06)	<0.0001	
≥140	1.03 (1.02, 1.04)	<0.0001	
DBP (mmHg)			<0.0001
<90	1.05 (1.04, 1.05)	<0.0001	
≥90	1.01 (1.00, 1.03)	0.0540	
Family history of diabetes			0.9508
No	1.04 (1.03, 1.05)	<0.0001	
Yes	1.04 (1.01, 1.07)	0.0078	

The model was adjusted to account for baseline BMI, SBP, DBP, ALT, TC, TG, HDL-c, LDL-c, BUN, Cr, smoking habits, alcohol consumption, diabetes family history, and FPG. For every instance, the model remains unadjusted concerning the stratification variable when it was categorical.

## Discussion

In this retrospective cohort study, we investigated the association between BF% and diabetes incidence among Chinese patients. We found that a 1% increase in BF% was associated with a 1.04-fold higher risk of diabetes (HR: 1.04, 95% CI: 1.03–1.05, *p* < 0.0001). Sensitivity and subgroup analyses further confirmed the robustness and reliability of these findings. Additionally, we identified a threshold effect, revealing a nonlinear relationship between BF% and diabetes incidence.

Obesity is widely recognized as a critical risk factor for T2DM (22, 23). BMI has become the most commonly used anthropometric indicator for diagnosing obesity and predicting diabetes due to its simplicity in measurement and calculation (24). However, in individuals with normal BMI who experience muscle loss and high body fat, using BMI to assess obesity can underestimate its prevalence (25). In older adults, BMI can also be overestimated due to height loss from aging and the presence of sarcopenia (25, 26). Moreover, BMI is influenced by factors such as gender, age, race, and short stature, which can distort body fat estimation. Research (27) have shown many diabetic patients have a normal BMI, but their body fat distribution is notably abnormal. BF% represents the proportion of body weight composed of fat and serves as a crucial indicator of health status and body composition (28). It provides a more accurate assessment of body fat compared to BMI and has shown a strong association with diabetes risk. A study (29) evaluated the relationship between BF% and the risk of developing T2DM in 5,972 Korean adults over 10 years. The results indicated that the risk of T2DM increased significantly when BF% exceeded 22.8% in men and 32.9% in women, with non-obese men showing a lower threshold (22.8%) compared to obese men (28.4%). Furthermore, men with a high BF% (≥25) had 1.83 times the risk of developing diabetes compared to those with a low BMI (<25), while no significant association was observed in women in the same subgroup. Another study (14) of 5,595 Chinese adults aged 18–65 from the China Health and Nutrition Survey (CHNS) in 2015 and 2018 found that the risk of T2DM increased significantly with higher BF%. Males with trunk BF% over 25.5% and females with trunk BF% over 34.4% were at a notably higher risk of developing diabetes.

Our study reached similar conclusions using a multivariate Cox proportional hazards regression model, which showed that a 1% increase in BF% was associated with a 1.04-fold higher risk of diabetes (HR: 1.04, 95% CI: 1.03–1.05, *p* < 0.0001). The risk of diabetes increased progressively across BF% quartiles (Q1 to Q4), with Q4 showing a significantly higher risk than Q1 (adjusted HR = 2.72, 95% CI = 2.19–3.37). These findings emphasize a strong positive association between higher BF% and increased diabetes risk. The consistency across models, even after adjusting for various confounders, highlights BF% as a crucial factor in diabetes risk management. Additionally, we revealed the nonlinear relationship between BF% and diabetes risk. The results of the analysis using the piecewise Cox proportional hazards regression model showed that the inflection point of BF% was 25.09%. Our study found that among individuals with a BF% below 25.09%, the risk of diabetes increased significantly with each 1-unit increase in BF%. However, when the BF% was above 25.09%, the increase in diabetes risk became more gradual with increasing BF%. The possible reasons are as follows: First, in the population with a BF% of 25.09% or higher, BMI and blood lipids (TC, TG, LDL-c) were significantly increased, but HDL-c was even higher, suggesting adaptive lipid metabolism adjustments (such as enhanced reverse cholesterol transport) that partially offset the metabolic risks. Second, in the population with a BF% of 25.09% or higher, FBG and blood pressure (SBP/DBP) were significantly elevated, and age also increased, consistent with the pathological characteristics of insulin resistance and cardiovascular risks (Supplementary Table S2). To further analyze gender differences, we conducted stratified analyses and found that the relationship between BF% and diabetes risk was nonlinear in both males and females, with inflection points at 24.60% for males and 34.93% for females, showing similar trends. These findings imply that clinicians can use these thresholds for personalized diabetes risk assessment. The differing thresholds between genders also highlight the need for gender-specific considerations in diabetes prevention and management. Overall, our results stress the importance of monitoring BF% and considering gender differences to better manage diabetes risk.

To the best of our knowledge, this is the largest cohort study to date investigating the link between BF% and diabetes risk. To ensure the robustness of the findings, we applied stratified Cox regression models for subgroup and sensitivity analyses. The subgroup analysis confirmed that the correlation remained consistent in individuals with a BMI lower than 25 kg/m2, systolic blood pressure below 140 mmHg, and diastolic blood pressure under 90 mmHg. Additionally, the subgroup analysis showed hazard ratios of 1.05 for individuals with a BMI under 25 and 1.04 for those with a BMI of 25 or higher (P for interaction >0.05), indicating that BF% is an independent risk factor regardless of BMI. Furthermore, the analysis showed that in non-overweight individuals, the risk of diabetes increased more with BF% (HR: 1.05 vs. 1.04). For leaner individuals, maintaining a healthy body fat percentage is crucial for diabetes prevention. Furthermore, the analysis showed that the risk of diabetes was higher in individuals under 60 compared to those over 60 (HR: 1.03 vs. 1.01), suggesting that younger individuals are more sensitive to increases in BF%. Therefore, screening for diabetes risk factors in individuals with normal BMI is crucial, particularly in younger populations, who should place greater emphasis on monitoring BF% to better assess their diabetes risk. Previous studies have reported significant differences in biological risk factors and pathophysiological mechanisms for diabetes between men and women (30). Our findings also support this conclusion, as women had a higher diabetes risk (HR: 1.19) than men (HR: 1.16), indicating gender differences in the influence of BF% on the pathogenesis of diabetes. This may be due to differences in fat distribution and endocrine hormone secretion between men and women. Therefore, further research is needed to explore the impact of BF% on diabetes risk factors in different genders and the underlying mechanisms.

Insulin resistance and impaired insulin secretion are the primary mechanisms in the development of T2DM (31). While obesity is closely linked to insulin resistance and the onset of diabetes, not all obese individuals with insulin resistance progress to diabetes (32), suggesting that other mechanisms are involved in the impact of BF% on diabetes risk. Obesity not only increases total body fat but also affects fat distribution, with visceral fat playing a particularly crucial role in metabolic health and disease risk. Visceral fat is more sensitive to lipolytic stimulation, leading to the release of large amounts of free fatty acids (FFAs) into the bloodstream. These FFAs are then deposited as ectopic fat in organs such as the liver, muscles, and pancreas, which impairs insulin sensitivity and affects insulin production (13). Additionally, an increase in visceral fat disrupts adipocyte function, altering the secretion of hormones such as leptin, resistin, and adiponectin, as well as various cytokines (33). The imbalance in these hormone levels plays a key role in the development of insulin resistance. Visceral fat also releases pro-inflammatory molecules like TNF-*α* and IL-6, which interfere with insulin signaling and worsen insulin resistance (34, 35). This inflammatory response, combined with the accumulation of FFAs, further increases insulin resistance in key tissues such as muscles and the liver, exacerbating hyperglycemia.

Our study had several strengths: Firstly, the total sample size was relatively large. To the best of our knowledge, this research represents the largest cohort study to date investigating the link between BF% and diabetes risk. Secondly, the study uncovered a nonlinear relationship between BF% and diabetes risk, with Cox proportional hazards regression identifying a critical BF% threshold that significantly impacts diabetes risk. Thirdly, the BF% in this study was derived from height, weight, age, and gender, making it easily obtainable with minimal error, which is suitable for wide-scale promotion. Additionally, a comprehensive range of sensitivity tests was performed, including subgroup evaluations and the use of generalized additive models to treat continuous covariates as smooth terms. The findings were further validated in individuals under 25 kg/m^2^ with systolic blood pressure below 140 mmHg and diastolic blood pressure under 90 mmHg, underscoring the robustness and reliability of the study’s conclusions.

The potential limitations of this study are as follows. First, the study focused solely on Chinese participants, which limits the generalizability of the findings to other populations. To address this issue, we plan to analyze data from regions like Europe, America, Japan, and Korea to investigate the global relationship between BF% and diabetes risk. Our goal is to develop population-specific prevention strategies and refine risk-assessment models for diverse groups. Second, although we adjusted for known confounding factors, unmeasured or uncontrolled variables could still have influenced the results. Third, as a secondary analysis of an existing database, the study lacked certain important variables that may have impacted the conclusions. Fourth, the diabetes diagnosis relied on FPG levels and self-reported data, potentially underestimating diabetes prevalence, as more precise methods like the 2-h oral glucose tolerance test or glycated hemoglobin were not used. Fifth, BF% and other parameters were measured only at baseline, missing any longitudinal changes that could provide deeper insights. Sixth, while there have been studies in China on the relationship between BF% and diabetes, they had small sample sizes and used different methods for measuring BF%, highlighting a fundamental distinction from this study. Seventh, we recognize that the accuracy of the BFP calculated by this formula needs validation against gold standard methods, such as DEXA. To enhance the reliability of our study, we suggest that future research conduct comparative analyses between this formula and gold standard measurements to assess its accuracy. Lastly, the retrospective, observational design of the study allows us to identify associations between BF% and diabetes risk but does not establish causality. Future prospective studies with diverse populations and comprehensive, longitudinal data are needed to better understand these relationships and the underlying mechanisms.

## Conclusion

This study highlights that BF% is a significant predictor of diabetes risk and reveals a positive, nonlinear relationship between BF% and diabetes risk in Chinese adults. Reducing BF% below the identified threshold could substantially reduce the likelihood of developing diabetes.

## Data Availability

The original contributions presented in the study are included in the article/Supplementary material, further inquiries can be directed to the corresponding authors.

## Footnotes

^1^ www.datadryad.org

## References

[1] Kong D Duan Y Wang J Liu Y. A functional polymorphism of microRNA-143 is associated with the risk of type 2 diabetes mellitus in the northern Chinese Han population. Front Endocrinol. (2022) 13:994953. doi: 10.3389/fendo.2022.994953, PMID: 36213264 PMC9538736

[2] Sun Y Wang Z Huang Z Hu H Han Y. The association between the triglyceride-to-high-density lipoprotein cholesterol ratio and the risk of progression to diabetes from prediabetes: a 5-year cohort study in Chinese adults. Front Endocrinol. (2022) 13:947157. doi: 10.3389/fendo.2022.947157, PMID: 35923622 PMC9340202

[3] Wang T Huang T Li Y Zheng Y Manson JAE Hu FB. Low birthweight and risk of type 2 diabetes: a Mendelian randomisation study. Diabetologia. (2016) 59:1920–7. doi: 10.1007/s00125-016-4019-z, PMID: 27333884 PMC4970938

[4] Cho NH Shaw JE Karuranga S Huang Y da Rocha Fernandes JD Ohlrogge AW. IDF diabetes atlas: global estimates of diabetes prevalence for 2017 and projections for 2045. Diabetes Res Clin Pract. (2018) 138:271–81. doi: 10.1016/j.diabres.2018.02.023, PMID: 29496507

[5] Jørgensen JT Bräuner EV Backalarz C Laursen JE Pedersen TH Jensen SS. Long-term exposure to road traffic noise and incidence of diabetes in the Danish nurse cohort. Environ Health Perspect. (2019) 127:57006. doi: 10.1289/EHP4389, PMID: 31084449 PMC6791549

[6] Carlsson S Andersson T Talbäck M Feychting M. Incidence and prevalence of type 2 diabetes by occupation: results from all Swedish employees. Diabetologia. (2020) 63:95–103. doi: 10.1007/s00125-019-04997-5, PMID: 31570970 PMC6890587

[7] Kim S Kyung C Park JS Lee SP Kim HK Ahn CW. Normal-weight obesity is associated with increased risk of subclinical atherosclerosis. Cardiovasc Diabetol. (2015) 14:58. doi: 10.1186/s12933-015-0220-5, PMID: 25990248 PMC4488951

[8] Dieli-Conwright CM Wong L Waliany S Bernstein L Salehian B Mortimer JE. An observational study to examine changes in metabolic syndrome components in patients with breast cancer receiving neoadjuvant or adjuvant chemotherapy. Cancer. (2016) 122:2646–53. doi: 10.1002/cncr.30104, PMID: 27219902 PMC4992442

[9] Du S Hong X Yang Y Ding Z Yu T. Association between body fat percentage and H-type hypertension in postmenopausal women. Front Public Health. (2022) 10:950805. doi: 10.3389/fpubh.2022.950805, PMID: 35937205 PMC9354540

[10] Dun Y Thomas RJ Medina-Inojosa JR Squires RW Huang H Smith JR. High-intensity interval training in cardiac rehabilitation: impact on fat mass in patients with myocardial infarction. Mayo Clin Proc. (2019) 94:1718–30. doi: 10.1016/j.mayocp.2019.04.033, PMID: 31486378 PMC6755673

[11] Han TS Al-Gindan YY Govan L Hankey CR Lean MEJ. Associations of BMI, waist circumference, body fat, and skeletal muscle with type 2 diabetes in adults. Acta Diabetol. (2019) 56:947–54. doi: 10.1007/s00592-019-01328-3, PMID: 30927105 PMC6597601

[12] Escobedo-de la Peña J Ramírez-Hernández JA Fernández-Ramos MT González-Figueroa E Champagne B. Body fat percentage rather than body mass index related to the high occurrence of type 2 diabetes. Arch Med Res. (2020) 51:564–71. doi: 10.1016/j.arcmed.2020.05.010, 32482372 32482372

[13] Gómez-Ambrosi J Silva C Galofré JC Escalada J Santos S Gil MJ. Body adiposity and type 2 diabetes: increased risk with a high body fat percentage even having a normal BMI. Obesity (Silver Spring). (2011) 19:1439–44. doi: 10.1038/oby.2011.36, PMID: 21394093

[14] Zhang S Jiang H Wang L Jia X Zhang J Wang H. Longitudinal relationship between body fat percentage and risk of type 2 diabetes in Chinese adults: evidence from the China health and nutrition survey. Front Public Health. (2022) 10:1032130. doi: 10.3389/fpubh.2022.1032130, PMID: 36523583 PMC9744757

[15] Chen Y Zhang Y Yuan J Zhang XP Cai B Wang XL. Association of body mass index and age with incident diabetes in Chinese adults: a population-based cohort study. BMJ Open. (2018) 8:e021768. doi: 10.1136/bmjopen-2018-021768, PMID: 30269064 PMC6169758

[16] Deurenberg P Weststrate JA Seidell JC. Body mass index as a measure of body fatness: age- and sex-specific prediction formulas. Br J Nutr. (1991) 65:105–14. doi: 10.1079/bjn19910073, PMID: 2043597

[17] He X Zhu Z Zang J Wang Z Liao P Wang W. Percent body fat, but not body mass index, is associated with cardiometabolic risk factors in children and adolescents. Chronic Dis Transl Med. (2023) 9:143–53. doi: 10.1002/cdt3.54 37305104 PMC10249195

[18] American Diabetes Association Professional Practice Committee. 2. Diagnosis and classification of diabetes: standards of Care in Diabetes-2024. Diabetes Care. (2024) 47:S20–42. doi: 10.2337/dc24-S002, PMID: 38078589 PMC10725812

[19] Huang Z Han Y Hu H Cao C Liu D Wang Z. Triglyceride to high-density lipoprotein cholesterol ratio is associated with regression to normoglycemia from prediabetes in adults: a 5-year cohort study in China. J Transl Med. (2023) 21:868. doi: 10.1186/s12967-023-04752-w, PMID: 38037094 PMC10688482

[20] von Elm E Altman DG Egger M Pocock SJ Gøtzsche PC Vandenbroucke JP. The strengthening the reporting of observational studies in epidemiology (STROBE) statement: guidelines for reporting observational studies. Lancet. (2007) 370:1453–7. doi: 10.1016/S0140-6736(07)61602-X 18064739

[21] Zheng X Zhang X Han Y Hu H Cao C. Nonlinear relationship between atherogenic index of plasma and the risk of prediabetes: a retrospective study based on Chinese adults. Cardiovasc Diabetol. (2023) 22:205. doi: 10.1186/s12933-023-01934-0, PMID: 37563588 PMC10416492

[22] Ge Q Xie X Chen X Huang R Rui CX Zhen Q. Circulating exosome-like vesicles of humans with nondiabetic obesity impaired islet β-cell proliferation, which was associated with decreased Omentin-1 protein cargo. Genes Dis. (2022) 9:1099–113. doi: 10.1016/j.gendis.2020.12.011, PMID: 35685466 PMC9170582

[23] Lee HA Cho JH Afinanisa Q An GH Han JG Kang HJ. Ganoderma lucidum extract reduces insulin resistance by enhancing AMPK activation in high-fat diet-induced obese mice. Nutrients. (2020) 12:3338. doi: 10.3390/nu12113338, PMID: 33142995 PMC7693844

[24] Zhao L Huang H Wang Y Zhao LL Wang TB Zhou H. Lifestyle factors and long-term survival of gastric cancer patients: a large bidirectional cohort study from China. World J Gastroenterol. (2020) 26:1613–27. doi: 10.3748/wjg.v26.i14.1613, PMID: 32327910 PMC7167420

[25] Lin CL Yu NC Wu HC Lee YY Lin WC Chiu IY. Association of Body Composition with type 2 diabetes: a retrospective chart review study. Int J Environ Res Public Health. (2021) 18:4421. doi: 10.3390/ijerph18094421, PMID: 33919339 PMC8122668

[26] Kompaniyets L Goodman AB Wiltz JL Shrestha SS Grosse SD Boehmer TK. Inpatient care cost, duration, and acute complications associated with BMI in children and adults hospitalized for COVID-19. Obesity. (2022) 30:2055–63. doi: 10.1002/oby.23522, PMID: 35730688 PMC9350354

[27] Xu S Ming J Jia A Yu X Cai J Jing C. Normal weight obesity and the risk of diabetes in Chinese people: a 9-year population-based cohort study. Sci Rep. (2021) 11:6090. doi: 10.1038/s41598-021-85573-z, PMID: 33731778 PMC7969601

[28] Luo E Zhang J Song J Feng D Meng Y Jiang H. Serum anti-Müllerian hormone levels were negatively associated with body fat percentage in PCOS patients. Front Endocrinol (Lausanne). (2021) 12:659717. doi: 10.3389/fendo.2021.659717, PMID: 34149614 PMC8213015

[29] Park SK Ryoo JH Oh CM Choi JM Jung JY. Longitudinally evaluated the relationship between body fat percentage and the risk for type 2 diabetes mellitus: Korean genome and epidemiology study (KoGES). Eur J Endocrinol. (2018) 178:513–21. doi: 10.1530/EJE-17-0868, PMID: 29523634

[30] Voigt S van Os H van Walderveen M van der Schaaf I Kappelle LJ Broersen A. Sex differences in intracranial and extracranial atherosclerosis in patients with acute ischemic stroke. Int J Stroke. (2021) 16:385–91. doi: 10.1177/1747493020932806, PMID: 32878572 PMC8193620

[31] Shimodaira M Niwa T Nakajima K Kobayashi M Hanyu N Nakayama T. Impact of serum triglyceride and high density lipoprotein cholesterol levels on early-phase insulin secretion in normoglycemic and prediabetic subjects. Diabetes Metab J. (2014) 38:294–301. doi: 10.4093/dmj.2014.38.4.294, PMID: 25215276 PMC4160583

[32] Kahn SE Hull RL Utzschneider KM. Mechanisms linking obesity to insulin resistance and type 2 diabetes. Nature. (2006) 444:840–6. doi: 10.1038/nature05482, PMID: 17167471

[33] Ouchi N Parker JL Lugus JJ Walsh K. Adipokines in inflammation and metabolic disease. Nat Rev Immunol. (2011) 11:85–97. doi: 10.1038/nri2921, PMID: 21252989 PMC3518031

[34] Hoene M Weigert C. The role of interleukin-6 in insulin resistance, body fat distribution and energy balance. Obes Rev. (2008) 9:20–9. doi: 10.1111/j.1467-789X.2007.00410.x, PMID: 17956545

[35] Hotamisligil GS. The role of TNFalpha and TNF receptors in obesity and insulin resistance. J Intern Med. (1999) 245:621–5. doi: 10.1046/j.1365-2796.1999.00490.x, PMID: 10395191

