# Supplementary material for: Elevated body fat percentage is linked to increased risk of diabetes: a longitudinal retrospective cohort study based on Chinese adults
Source: Front Nutr. 2025 Jun 19;12:1510210. doi: 10.3389/fnut.2025.1510210 (PMC12221890; doi:10.3389/fnut.2025.1510210)
Supplement: Supplementary file 1 [file Data_Sheet_1.docx]

**Elevated body fat percentage is linked to increased risk of diabetes:** **A longitudinal retrospective cohort study based on Chinese adults**

Weicong Pan^1^, Shichun Cai^2^, Zhenhua Huang^1*^ and Ke Yu ^3*^

1 Department of Emergency Medicine, the First Affiliated Hospital of Shenzhen University, Shenzhen Second People’s Hospital, Shenzhen, 518035, China.

2 Quality Control Department and Preventive Health Department, Pengpai Memorial Hospital, Shanwei, 516499, China.

3 Department of Pulmonary and Critical Care Medicine, the First Affiliated Hospital of Shenzhen University, Shenzhen Second People’s Hospital, Shenzhen, 518035, China.

**Supplement Table 1** Relationship between BF% and risk of diabetes in different models in the imputed data.

| Exposure |  | Crude model (HR,95%CI) P | Model I(HR,95%CI) P | Model II(HR,95%CI) P |
| --- | --- | --- | --- | --- |
| BF% |  | 1.10 (1.10, 1.11) <0.0001 | 1.05 (1.05, 1.06) <0.0001 | 1.04 (1.04, 1.05) <0.0001 |
| (BF% quartiles) |  |  |  |  |
| Q1 |  | Ref | Ref | Ref |
| Q2 |  | 3.65 (3.13, 4.25) <0.0001 | 2.69 (2.31, 3.14) <0.0001 | 2.02 (1.73, 2.36) <0.0001 |
| Q3 |  | 5.90 (5.09, 6.83) <0.0001 | 3.89 (3.35, 4.52) <0.0001 | 2.60 (2.23, 3.03) <0.0001 |
| Q4 |  | 9.34 (8.09, 10.78) <0.0001 | 4.51 (3.88, 5.23) <0.0001 | 3.05 (2.60, 3.57) <0.0001 |
| P for trend |  | <0.0001 | <0.0001 | <0.0001 |

Crude model: we did not adjust other covariates.

Model I: we adjusted BMI.

Model II: we adjusted BMI, SBP, DBP, ALT, TC, TG, HDL-c, LDL-c, BUN, Cr, smoking and drinking habits, family history of diabetes, and FPG at baseline.

**Supplement Table 2:** The baseline characteristics of participants between the inflection point.

| BF% | ＜25.09% | ≥25.09% | P-value |
| --- | --- | --- | --- |
| participants | 92,855 | 118,978 |  |
| Age (years) | 36.37 ± 8.98 | 46.57 ± 13.29 | <0.001 |
| BMI (kg/m2) | 22.19 ± 2.81 | 24.05 ± 3.50 | <0.001 |
| SBP (mmHg) | 118.05 ± 14.16 | 119.86 ± 17.88 | <0.001 |
| DBP (mmHg) | 73.65 ± 9.83 | 74.59 ± 11.51 | <0.001 |
| FBG (mg/dL) | 4.85 ± 0.58 | 4.97 ± 0.63 | <0.001 |
| TC (mmol/L) | 4.57 ± 0.86 | 4.81 ± 0.92 | <0.001 |
| TG (mmol/L) | 1.30 ± 0.95 | 1.37 ± 1.09 | <0.001 |
| HDL-c (mmol/L) | 1.34 ± 0.30 | 1.39 ± 0.31 | <0.001 |
| LDL-c (mmol/L) | 2.69 ± 0.65 | 2.82 ± 0.70 | <0.001 |
| Gender, n (%) |  |  | <0.001 |
| Male | 51763 (97.77%) | 34644 (65.47%) |  |
| Female | 1182 (2.23%) | 18275 (34.53%) |  |

Continuous variables were summarized as mean (SD) or medians (quartile interval); categorical variables were displayed as percentage (%). Abbreviations: BF%, body fat percentage; BMI, body mass index; SBP, systolic blood pressure; DBP; diastolic blood pressure; TC, total cholesterol; TG triglyceride; HDL-c, high-density lipoprotein cholesterol; ALT, alanine aminotransferase; FBG, fasting plasma glucose.


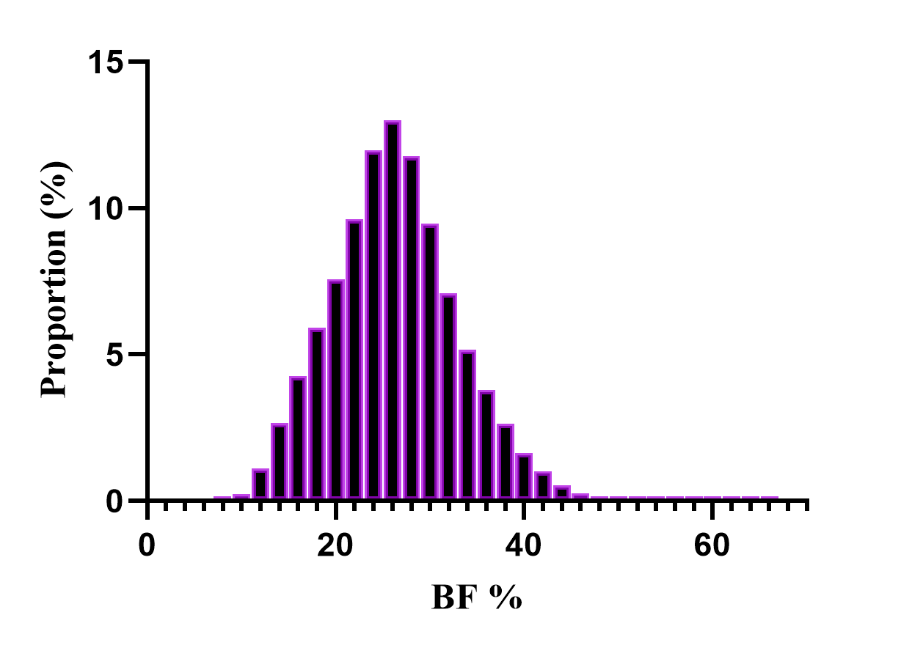


**Supplement Figure. 1** Distribution of BF%. It presented a normal distribution, ranging from 7.59 to 66.89, with a mean of 26.25.


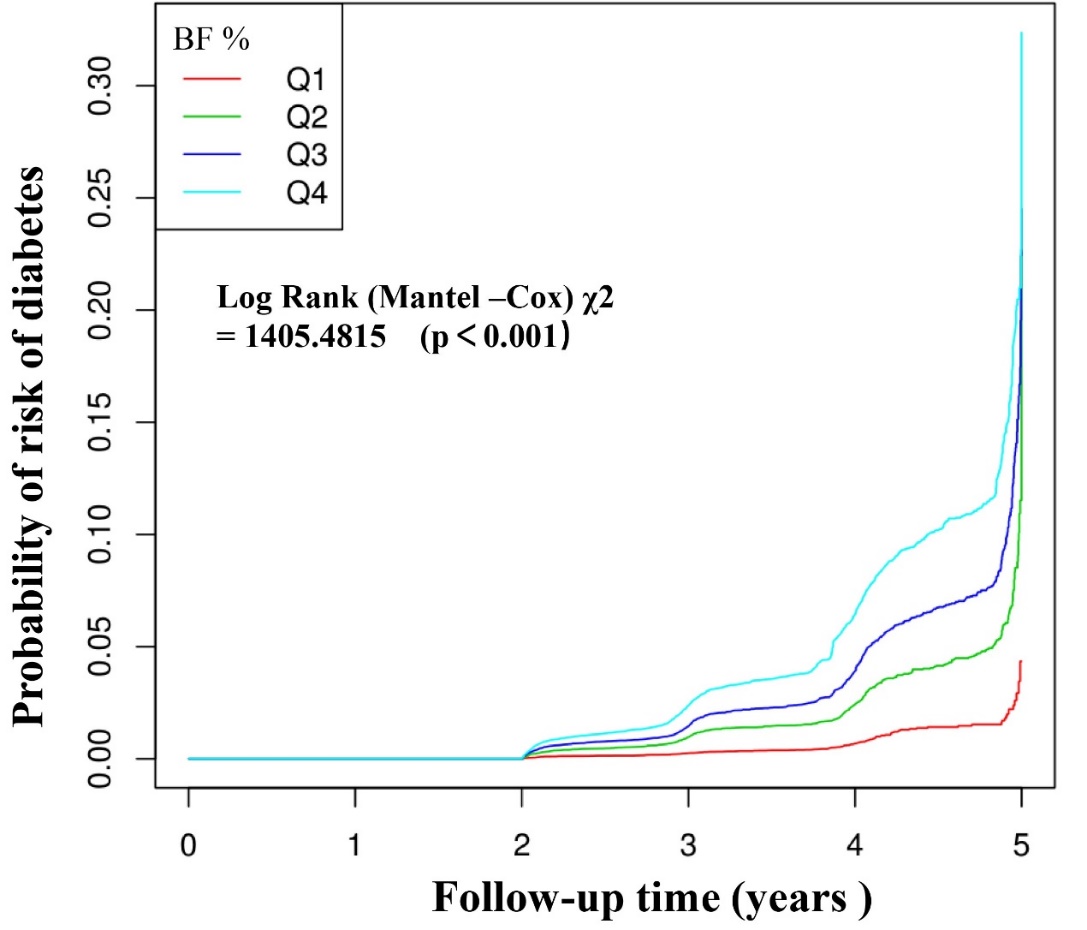


**Supplement Figure. 2** Kaplan–Meier curves for the probability of diabetes. The probability of diabetes increased progressively with rising BF%, meaning that patients with the highest BF% had the higher probability of diabetes.
